# Post-stress left ventricular ejection fraction drop in patients with diabetes: a gated myocardial perfusion imaging study

**DOI:** 10.1186/1471-2261-13-99

**Published:** 2013-11-14

**Authors:** Adele Ferro, Mario Petretta, Wanda Acampa, Giovanni Fiumara, Stefania Daniele, Maria Piera Petretta, Valeria Cantoni, Alberto Cuocolo

**Affiliations:** 1 Institute of Biostructure and Bioimaging, National Council of Research, Napoli, Italy; 2 Department of Translational Medical Sciences, University Federico II, Napoli, Italy; 3 SDN Foundation, Institute of Diagnostic and Nuclear Development, Napoli, Italy; 4 Department of Advanced Biomedical Sciences, University Federico II, Napoli, Italy

**Keywords:** Diabetes mellitus, Gated myocardial perfusion imaging, Left ventricular ejection fraction, Myocardial stunning

## Abstract

**Background:**

To evaluate the relevance of stress-induced decrease in left ventricular ejection fraction (LVEF) in patients with type-2 diabetes.

**Methods:**

A total of 684 diabetic patients with available rest and post-stress gated myocardial perfusion single-photon emission computed tomography (MPS) data were enrolled. An automated algorithm was used to determine the perfusion scores using a 17-segment model. LVEF drop was considered significant if the post-stress LVEF was ≥5% below the rest value. Follow-up data were available in 587 patients that were followed for the occurrence of cardiac death, nonfatal myocardial infarction, or unstable angina requiring revascularization.

**Results:**

A post-stress LVEF drop ≥5% was observed in 167 (24%) patients. Patients with LVEF drop had higher summed stress score (p < 0.05), summed difference score (p < 0.001), and rest LVEF (p < 0.001) compared to patients without. Conversely, summed rest score, a measure of infarct size, was comparable between the two groups. At multivariable analysis, summed difference score and rest LVEF were independent predictors (both p < 0.001) of post-stress LVEF drop. Myocardial perfusion was abnormal in 106 (63%) patients with post-stress LVEF drop and in 296 (57%) of those without (p = 0.16). The overall event-free survival was lower in patients with post-stress LVEF drop than in those without (log rank χ^2^ 7.7, p < 0.005). After adjusting for clinical data and MPS variables, the hazard ratio for cardiac events for post-stress LVEF drop was 1.52 (p < 0.01).

**Conclusions:**

In diabetic patients stress-induced ischemia is an independent predictor of post-stress LVEF drop; however, a reduction in LVEF is detectable also in patients with normal perfusion. Finally, post-stress LVEF drop increases the risk of subsequent cardiac events in diabetic patients.

## Background

Braunwald and Kloner [1] originally described myocardial stunning as ‘delayed recovery of regional myocardial contractile function after reperfusion despite the absence of irreversible damage and despite restoration of normal flow’. Stunning may be manifested on gated myocardial perfusion single-photon emission computed tomography (MPS) as wall motion abnormalities or as a post-stress decrease in left ventricular ejection fraction (LVEF) [2-7]. A drop in post-stress LVEF is an additional sign of coronary artery disease (CAD) severity [8] and a prognostic marker of cardiovascular events [9]. It is also known that CAD is more prevalent and severe in patients with diabetes mellitus and the association between diabetes and CAD is increasingly better understood [10-12]. Emerging data support the utility of stress imaging in identifying diabetic patients with preclinical CAD [13]. Diabetic patients have high incidence of heart failure [14,15] and recognition of myocardial stunning may be useful in these patients [16]. Despite an extensive use of stress MPS, no study specifically addressed the significance of a drop in post-stress LVEF in diabetic patients. The aim of this study was to assess the relevance of post-stress LVEF drop as evaluated by gated MPS in a large cohort of diabetic patients.

## Methods

### Patients

The study population included 684 (461 men, age 63 ± 9 years) consecutive patients with type-2 diabetes and available rest and stress gated MPS data, referred from October 2005 to May 2007 for MPS for the detection of myocardial ischemia. Among the overall patient population, 74% had hypertension, 57% dyslipidemia, 34% family history of CAD, and 39% history of myocardial infarction. Patients have been excluded from study for: 1) recurrent chest pain unresponsive to anti-ischemic medications; 2) recent acute coronary syndrome, stroke, or transient ischemic attack (last 3 months); 3) uncompensated congestive heart failure (New York Heart Association class III or IV) or recent admission for congestive heart failure (last 3 months); 4) prior myocardial revascularization procedures; 5) an absolute contraindication to dipyridamole in subjects with inability to exercise; or 6) a concomitant noncardiac illness. The ethics committee for the Biomedical Activities of the University Federico II approved the study and all patients gave informed consent.

### Gated MPS

All patients underwent same-day Tc-99 m sestamibi rest and stress gated MPS by exercise or dipyridamole stress test, according to the recommendations of the European Association of Nuclear Medicine and European Society of Cardiology [17]. An automated software program was used to calculate LV volumes and EF and the scores incorporating both the extent and severity of perfusion defects, using standardized segmentation of 17 myocardial regions [18]. The difference between the post-stress and rest LVEF was calculated. A significant LVEF reduction was defined as a drop ≥5% between the post-stress and rest LVEF [3,19]. Each myocardial segment was scored from normal (score = 0) to absent perfusion (score = 4). The summed stress score was obtained by adding the scores of the 17 segments of the stress images. A similar procedure was applied to the resting images to calculate the summed rest score. The summed difference score represents the difference between the stress and rest scores and is taken to be an index of ischemic burden. Patients were considered to have an abnormal MPS with a summed stress score >3. Significant ischemia was defined by a summed difference score >2, and classified as mild/moderate (2 to 6) and severe (>6) [20].

### Coronary angiography

Of the 684 total patients included, 243 underwent coronary angiography within 3 months of MPS. Coronary angiography was performed using the standard Judkins method. Experienced cardiologists visually interpreted all coronary angiograms. Significant CAD was defined as luminal coronary diameter stenosis of >50% in at least one major coronary artery distribution [21].

### Follow-up

Follow-up data were obtained by the use of a questionnaire that was assessed by a phone call to all patients and/or general practitioners or cardiologists and by review of hospital records by individuals blinded to the patient’s test results. The end point was the occurrence of cardiac death, nonfatal myocardial infarction, or unstable angina requiring revascularization whichever occurred first. The date of the last examination or consultation was used to determine the length of follow-up.

### Statistical analysis

Continuous variables are described as mean ± standard deviation and categorical data as percentages. Groups were compared using *t* test, the Fisher’s exact test, or χ^2^ test, as appropriate. A p value <0.05 was considered statistically significant. Univariable associations with post-stress LVEF drop ≥5% were determined by logistic regression analysis. A multivariable model was constructed using a stepwise regression strategy (p < 0.05 for model entry and p < 0.10 for model retention). To form this model patients’ age, sex, diabetes duration, hypertension, dyslipidemia, smoking, family history of CAD, history of myocardial infarction, stress type, and MPS variables were considered in the model selection process. Survival curves were constructed using the Kaplan-Meier method to account for censored survival times and were compared with the log rank test. A multivariable Cox proportional hazard regression model was built to calculate the hazard ratio of cardiac events considering patients’ clinical data, stress type, MPS variables, and post-stress LVEF drop ≥5%. The statistical software used was SPSS Inc., Advanced Models 15.0 (Chicago, Illinois).

## Results

**Figure 1:**
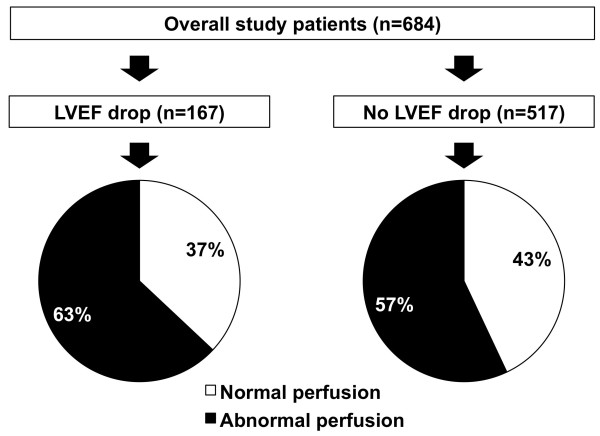
Pie graphs showing the proportions of patients with normal or abnormal myocardial perfusion according to the presence or absence of a post-stress LVEF drop ≥5%.

A total of 684 diabetic patients were included in the study. The mean LVEF was 56 ± 14% at rest and 55 ± 14% post-stress. Among the 684 diabetic patients included 167 had a post-stress reduction in LVEF ≥5%. The clinical characteristics and MPS findings of diabetic patients with and without post-stress LVEF drop are given in Table 1. As shown, summed stress score, summed difference score, and rest LVEF were significantly higher in patients with post-stress LVEF drop compared to those without. Conversely, rest end-systolic volume was significantly lower in patients with post-stress LVEF drop. Summed rest score, a measure of infarct size, and the clinical variables were comparable between the two groups. Myocardial perfusion was abnormal in 106 (63%) patients with post-stress LVEF drop and in 296 (57%) of those without (p = 0.16) (Figure 1). Noteworthy, 37% of patients with post-stress LVEF drop had normal myocardial perfusion.

**Table 1:** Clinical characteristics and MPS findings according to the presence or absence of a post-stress LVEF drop ≥5%

	**LVEF drop (n = 167)**	**No LVEF drop (n = 517)**	**p-value**
Age (years)	64 ± 10	63 ± 9	0.26
Male gender, *n* (%)	110 (66%)	351 (68%)	0.62
Diabetes duration (months)	127 ± 111	117 ± 112	0.35
Oral treatment, *n* (%)	92 (55%)	268 (52%)	0.47
Insulin and oral, *n* (%)	51 (31%)	165 (32%)	0.74
Insulin, *n* (%)	24 (14%)	84 (16%)	0.56
Statin, *n* (%)	32 (19%)	91 (18%)	0.65
Hypertension, *n* (%)	128 (77%)	381 (74%)	0.44
Dyslipidemia, *n* (%)	98 (59%)	293 (57%)	0.64
Smoking, *n* (%)	67 (40%)	210 (41%)	0.81
Family history of CAD, *n* (%)	56 (34%)	178 (34%)	0.83
Prior myocardial infarction, *n* (%)	70 (42%)	198 (38%)	0.58
Exercise stress test, *n* (%)	88 (53%)	292 (56%)	0.39
Summed stress score	8.2 ± 8.2	6.7 ± 7.4	<0.05
Summed rest score	3.9 ± 5.6	4.1 ± 5.9	0.55
Summed difference score	4.3 ± 5.1	2.5 ± 3.1	<0.001
Rest LVEF (%)	60 ± 12	54 ± 14	<0.001
Post-stress LVEF (%)	52 ± 12	55 ± 14	<0.01
Rest EDV (ml)	95 ± 39	101 ± 46	0.13
Post-stress EDV (ml)	98 ± 41	102 ± 46	0.37
Rest ESV (ml)	42 ± 27	50 ± 38	<0.05
Post-stress ESV (ml)	51 ± 32	50 ± 39	0.80

CAD: coronary artery disease, LVEF: left ventricular ejection fraction, EDV: end-diastolic volume, ESV: end-systolic volume.

### Predictors of post-stress LVEF drop

**Figure 2:**
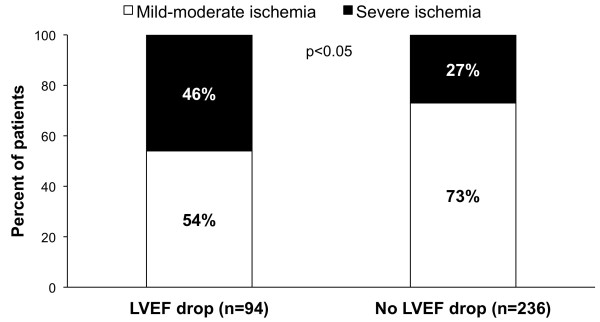
Bar graphs illustrating the relation between the magnitude of stress-induced ischemia and post-stress LVEF drop ≥5%.

**Figure 3:**
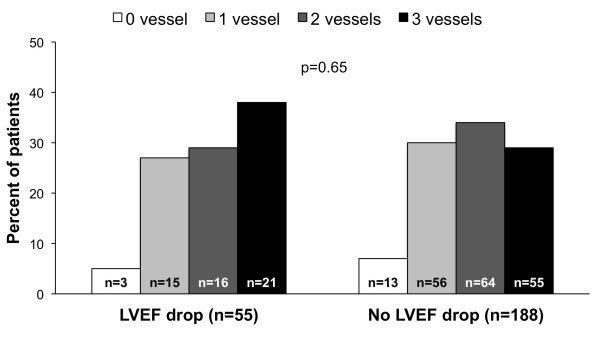
Distribution of coronary artery disease (number of diseased vessels) in relation to the presence or absence of a post-stress LVEF drop ≥5%.

Significant predictors of post-stress LVEF drop are reported in Table 2. As shown, at univariable analysis among all considered variables summed stress score, summed difference score, and LVEF at rest were significant predictors. At multivariate analysis, the only independent predictors were summed difference score and rest LVEF. The relation between the severity of ischemia and post-stress LVEF drop is illustrated in Figure 2. As shown, severe ischemia was more frequent in patients with post-stress LVEF drop than in those without (p < 0.05). In the subgroup of 243 patients who underwent coronary angiography, the distribution of the number of vessels with a significant coronary stenosis was similar in patients with and without of post-stress LVEF drop (Figure 3).

**Table 2:** Univariable and multivariable predictors of post-stress LVEF drop ≥5%

	**Univariable OR (95% CI)**	**p-value**	**Multivariable OR (95% CI)**	**p-value**
Summed stress score	1.02 (1.00-1.04)	<0.05		
Summed difference score	1.11 (1.06-1.16)	<0.001	1.16 (1.10-1.22)	<0.001
Rest LVEF	1.03 (1.02-1.05)	<0.001	1.05 (1.03-1.06)	<0.01

OR: odds ratio, CI: confidence interval, LVEF: left ventricular ejection fraction.

### Post-stress LVEF drop and outcome

**Figure 4:**
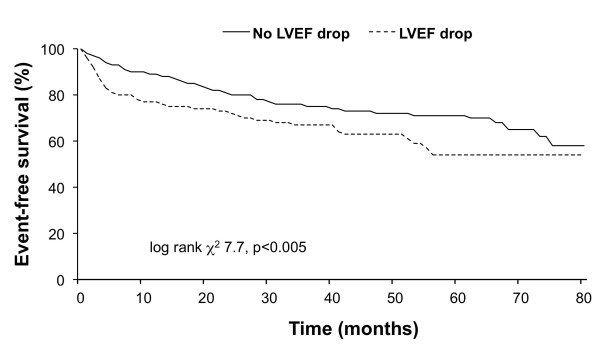
Event-free survival curves by Kaplan-Meier analysis according to the presence or absence of a post-stress LVEF drop ≥5%.

Follow-up data were available in 587 patients. The median follow-up was 51.6 months (interquartile range, 41.4-59.8). During follow-up, 181 end-point events occurred (31% cumulative event rate). The events were cardiac death in 41 patients, nonfatal myocardial infarction in 25 patients, and unstable angina requiring revascularization in 133 patients. The Kaplan-Meier analysis showed that the overall event-free survival was lower in patients with post-stress LVEF drop than in those without (log rank χ^2^ 7.7, p < 0.005) (Figure 4). The hazard ratio (95% confidence interval) for cardiac events for post-stress LVEF drop was 1.52 (1.10-2.11, p < 0.01), after adjusting for patients’ clinical data, stress type, and MPS variables. When only patients with normal myocardial perfusion were considered, event-free survival was comparable in patients with and without post-stress LVEF drop (log rank χ^2^ 2.5, p = 0.1).

## Discussion

To our knowledge, this is the first study addressing the relevance of post-stress LVEF drop in a large cohort of patients with type-2 diabetes. The results show that stress-induced ischemia is an independent predictor of a post-stress LVEF drop, but LVEF fall is also detectable in the absence of myocardial perfusion abnormalities. In contrast, neither clinical variables nor infarct size were associated with a post-stress LVEF drop.

It has been demonstrated that the post-stress LVEF drop in patients with CAD is linked to regional perfusion defects and predicts the presence of severe disease [3,22]. In diabetic patients an abnormal LVEF response to exercise has been documented by echocardiography or radionuclide angiography also in the absence of CAD [23-25]. Accumulating data showed that diabetes mellitus leads to cardiac functional and structural changes, independent on hypertension, CAD, or any other known cardiac disease, supporting the existence of a diabetic cardiomyopathy [26].

In the present study performed in diabetic patients, although severe ischemia was more frequent in patients with post-stress LVEF drop, the distribution of the number of diseased vessels was similar in patients with and without of post-stress LVEF drop. Noteworthy, post-stress LVEF drop was present in a substantial number of subjects (37%) without ischemia. Abnormality in the contractile response during stress might explain this finding, suggesting loss of contractile reserve [24]. An important epidemiological evidence of the independent effect of diabetes on LV systolic function is given by the results of the Strong Heart Study [27]. Compared with non-diabetics, patients with diabetes had greater LV mass, and lower LV fractional shortening after adjusting for confounding covariables [27]. In addition, the presence of post-stress LVEF drop in diabetic patients with normal perfusion may be also related to coronary vascular dysfunction in the absence of significant coronary artery stenosis [28-30]. This hypothesis is supported by the observation that in the subgroup of patients who underwent coronary angiography, the distribution of the number of vessels with a significant coronary stenosis was similar in patients with and without post-stress LVEF drop.

As expected, summed difference score was an independent predictor of post-stress LVEF drop. In particular, severe myocardial ischemia was found in 46% of patients with and in 27% of those without post-stress LVEF drop. Several studies reported that in patients with suspected or known CAD stress-induced transient LV dysfunction is associated with severe and extensive ischemia [3-7,31,32]. However, these studies evaluated post-stress LVEF drop in unselected patients population. The finding of a higher LVEF at rest in patients with post-stress LVEF drop is in agreement with previous studies. In particular, Guenancia et al. [33] in patients with recent myocardial infarction found high LVEF resting values as independent predictor of post-stress LVEF drop. In our study, patients with post-stress LVEF drop had a poorer outcome than those without. However, when only patients with normal myocardial perfusion were considered, event-free survival was comparable in patients with and without post-stress drop in LVEF, confirming the prognostic role of stress-induced ischemia.

This study has some potential limitations. First, perfusion patterns might influence the decrease of post-stress LVEF [34,35]. However, it has been demonstrated that gated MPS provides an accurate assessment of LVEF even in the presence of large perfusion defects as compared to equilibrium radionuclide angiography and echocardiography [36]. Another limitation of this study is the lack of hemoglobin A1c levels, which was not available in all patients. In addition, coronary angiography was not performed in all patients.

## Conclusions

In patients with diabetes stress-induced ischemia is an independent predictor of post-stress LVEF drop; however, a fall in LVEF is detectable also in patients with normal myocardial perfusion. These findings suggest that a post-stress LVEF drop may be related to a specific diabetic cardiomyopathy in the absence of myocardial perfusion abnormalities. Finally, post-stress LVEF drop increases the risk of subsequent cardiac events in diabetic patients.

## Abbreviations

CAD: Coronary artery disease; LVEF: Left ventricular ejection fraction; MPS: Myocardial perfusion single-photon emission computed tomography.

## Competing interests

The authors declare that they have no competing interests.

## Authors’ contributions

AF performed the statistical analysis and drafted the manuscript. SD made substantial contribution with statistical analysis. MP and AC contributed with the conception and design of the study. GF, MPP and VC analyzed the collected data. MP, WA, and AC participated in the study design and interpretation and revised the manuscript critically for important intellectual content. All authors revised the manuscript. All authors read and approved the final manuscript.

## Pre-publication history

The pre-publication history for this paper can be accessed here:

http://www.biomedcentral.com/1471-2261/13/99/prepub
